# Contingency management to reduce methamphetamine use and sexual risk among men who have sex with men: a randomized controlled trial

**DOI:** 10.1186/1471-2458-10-774

**Published:** 2010-12-20

**Authors:** Timothy W Menza, Damon R Jameson, James P Hughes, Grant N Colfax, Steven Shoptaw, Matthew R Golden

**Affiliations:** 1 Center for AIDS and STD, Harborview Medical Center, 325 Ninth Avenue, Seattle, WA, 98104, USA; 2 Department of Epidemiology, University of Washington School of Public Health, Box 357230, Seattle, WA, 98195, USA; 3 Department of Biostatistics, University of Washington School of Public Health, Box 357230, Seattle, WA, 98195, USA; 4 Department of Medicine, University of Washington School of Medicine, Box 356420, Seattle, WA, 98195, USA; 5 Public Health--Seattle & King County, 401 5th Ave., Suite 1300, Seattle, WA, 98104, USA; 6 San Francisco Department of Public Health, 101 Grove Street, Room 408, San Francisco, CA, 94102, USA; 7 Department of Family Medicine, David Geffen School of Medicine at UCLA, 50-078 Center for Health Sciences, Los Angeles, CA, 90095, USA; 8 Department of Psychiatry and Biobehavioral Sciences, David Geffen School of Medicine at UCLA, 760 Westwood Plaza, Los Angeles, CA, 90095, USA

## Abstract

**Background:**

Methamphetamine use is associated with HIV acquisition and transmission among men who have sex with men (MSM). Contingency management (CM), providing positive reinforcement for drug abstinence and withholding reinforcement when abstinence is not demonstrated, may facilitate reduced methamphetamine use and sexual risk. We compared CM as a stand-alone intervention to a minimal intervention control to assess the feasibility of conducting a larger, more definitive trial of CM; to define the frequency of behavioral outcomes to power such a trial; and, to compute preliminary estimates of CM's effectiveness.

**Methods:**

We randomly assigned 127 MSM from Seattle, WA who use methamphetamine to receive a 12-week CM intervention (n = 70) or referral to community resources (n = 57).

**Results:**

Retention at 24 weeks was 84%. Comparing consecutive study visits, non-concordant UAI declined significantly in both study arms. During the intervention, CM and control participants were comparably likely to provide urine samples containing methamphetamine (adjusted relative risk [aRR] = 1.09; 95%CI: 0.71, 1.56) and to report non-concordant UAI (aRR = 0.80; 95%CI: 0.47, 1.35). However, during post-intervention follow-up, CM participants were somewhat more likely to provide urine samples containing methamphetamine than control participants (aRR = 1.21; 95%CI: 0.95, 1.54, *P *= 0.11). Compared to control participants, CM participants were significantly more likely to report weekly or more frequent methamphetamine use and use of more than eight quarters of methamphetamine during the intervention and post-intervention periods.

**Conclusions:**

While it is possible to enroll and retain MSM who use methamphetamine in a trial of CM conducted outside drug treatment, our data suggest that CM is not likely to have a large, sustained effect on methamphetamine use.

**Trial Registration:**

ClinicalTrials.gov Identifier **NCT01174654**

## Background

Men who have sex with men (MSM) who report using methamphetamine are 1.5 to 2.9 times more likely to acquire HIV than men who do not [1-6]. MSM describe sex on methamphetamine as "automatic" and "compulsive" [7,8], and methamphetamine use has been consistently associated with sexual behaviors and biological factors that may facilitate HIV acquisition and transmission [9].

Reductions in sexual risk accompany participation in substance abuse treatment and peer-support programs [10,11]. In particular, contingency management (CM) has emerged as a promising intervention to reduce methamphetamine use and HIV-related sexual risk among MSM. CM is the practice of providing incentives for meeting a specified behavioral goal (e.g., abstinence from substance use) and withholding incentives when the goal is not met. Scientists have used CM to successfully promote an array of behavioral goals in diverse populations [12-14]. In a randomized trial that enrolled MSM seeking outpatient drug treatment, CM and cognitive behavioral therapies resulted in similar reductions in methamphetamine use and sexual risk [15]. The results of that trial, as well as data indicating high levels of interest in CM [16], prompted the San Francisco Department of Public Health to implement the Positive Reinforcement Opportunities Project (PROP), a stand-alone CM intervention among non-treatment-seeking MSM [17,18].

Despite evidence supporting the potential effectiveness of CM, the intervention's effects on methamphetamine use and sexual risk have not been tested using a minimal intervention control. We conducted a randomized controlled trial of CM to reduce methamphetamine use and sexual risk among MSM outside of drug treatment. The goals of the study were to assess the feasibility of conducting a larger, more definitive trial of CM; to define the frequency of behavioral outcomes to power such a trial; and, to compute preliminary estimates of CM's effectiveness.

## Methods

### Study population

We enrolled MSM from Seattle, WA. Between June 2007 and August 2008, participants were recruited through referrals from an STI clinic; an HIV clinic; community-based organizations (CBOs); printed media; radio; community outreach; and, referral by peers participating in the study.

This study was initially designed to inform the design of a trial to test whether CM could prevent HIV acquisition among HIV-negative men who use methamphetamine. Therefore, from June 2007 to November 2007, we enrolled only HIV-negative men. Because of slow enrollment of HIV-negative men, in November 2007, we opened the study to HIV-positive men and revised the study objective to test whether a CM intervention could reduce HIV acquisition and transmission risk. Several studies illustrate that a considerable proportion of HIV-positive men use methamphetamine and that methamphetamine facilitates HIV transmission behaviors [9]. Additional inclusion criteria were: age ≥18 years; willingness to be randomized and provide locator information; and, no plans to move from the study catchment area within 6 months of enrollment. Behavioral eligibility criteria were ≥1 episodes of anal sex and ≥2 episodes of methamphetamine use in the month prior to screening. We did not require a methamphetamine-positive urine sample for enrollment as we thought this requirement would limit the utility of CM as a public health intervention in practice. We excluded participants who reported a mutually monogamous relationship with a partner of the same HIV status lasting ≥2 years and men who expressly asked for detoxification, counseling, or drug rehabilitation services. HIV status was the only eligibility criterion modified during the course of the study.

### Study intervention and design

The study intervention was a 12-week CM program, adapted from previous studies, in which vouchers of escalating value were offered for consecutive urine samples that did not contain methamphetamine or crack or cocaine (herein referred to as cocaine) metabolites [15]. Initially, the intervention consisted of thrice-weekly drop-in urine testing visits for a total of 36 visits. Vouchers started at $2.50 for the first stimulant-free sample and increased by $1.25 for every consecutive stimulant-free sample thereafter up to a maximum of $10.00. Participants submitting three stimulant-free urine samples in a row earned a $10.00 bonus. The maximum payout for this program was $453.75, similar to the payout in other programs [17,18]. When participants submitted a stimulant-containing sample, or missed a visit, no vouchers were issued and the value of the voucher for the subsequent stimulant-free sample was reset to $2.50. If a participant submitted a week of stimulant-free samples after submitting a sample containing stimulant metabolites, he returned to the voucher value prior to the stimulant-containing sample ("rapid reset") [19]. Vouchers were redeemable immediately upon accrual for pre-paid gift cards and goods and services; we never offered cash. All CM participants signed a contract delineating the expectations of the CM program [20]. Study personnel administering the CM intervention followed a simple, scripted protocol for the reporting of results of urine testing and administering vouchers. Such a protocol was used to avoid the provision of counseling around the results of the urine testing. The protocol, which required no formal training, was developed by one of us (SS) who has extensive expertise in delivering CM interventions.

All seven participants enrolled in the study while the above procedures were in place reported difficulty adhering to the intervention schedule, and only two attended ≥12 of their 36 visits. In response, in September 2007, we reduced the number of weekly urine testing visits to two (24 visits over the 12-week intervention) and increased the value of vouchers for the first stimulant-free urine sample to $7.50; other studies have employed a similar schedule [21]. As before, these vouchers increased by $1.25 for each consecutive stimulant-free sample to a maximum of $10.00. Additionally, we gave participants a $20.00 bonus for two consecutive stimulant-free samples. The maximum payout for this program was $476.25. We continued to withhold vouchers and reset voucher values to baseline for urines containing stimulants and for missed visits; however, to encourage participants to attend visits, we gave men submitting stimulant-positive samples a voucher worth $2.50.

Under the initial CM intervention schedule, drop-in urine-testing visits were available from 10:00 am to 6:00 pm on Tuesdays, Thursdays, and Saturdays; we offered extended hours for working participants. After the enrollment visit, we sent postcards or e-mails to all participants encouraging participation in the intervention. We reminded participants who did not attend urine-testing visits for the first week by phone or e-mail. We sent postcards, phoned, or e-mailed all CM participants again at the midpoint of the intervention period. Under the revised CM intervention schedule, drop-in urine testing visits were available from 10:00 am to 6:00 pm on Tuesdays and Saturdays with flexible hours for working participants. Postcard, phone, and e-mail reminder strategies remained the same.

We tested urine samples with the QuickScreen Pro Multi-Drug Screening Test (Phamatech, Inc., San Diego, CA), a point-of-care test used to qualitatively detect stimulant metabolites. For this assay, the estimated mean detection time in urine ranges from 43.6 to 66.9 hours for methamphetamine [22] and is 88.4 hours for benzoylecgonine, a cocaine metabolite [23]. We repeated 10% of all urine tests; none were discordant. Study staff monitored the collection of all urine samples and tested the samples immediately after their provision.

Participants randomized to both control and CM arms received a printed list of local counseling, treatment, and outreach services at baseline and at each study visit. Study staff offered all participants assistance accessing services. Control participants did not submit twice-weekly urine samples and did not receive vouchers during the first 12 weeks of the study.

The study randomized participants using block sizes of two, four, and eight varied randomly with a pseudo-random number generator, a deterministic process use to generate an effectively unpredictable sequence of numbers [24]. The randomization list was used to assemble sequentially numbered, sealed, opaque envelopes containing intervention arm assignments. The research coordinator and principal investigator were blinded to the randomization code. From June 2007 to April 2008, participants were randomized 1:1 to the intervention and control arms under a protocol funded by the National Institute on Drug Abuse. In April 2008, we received additional funding from Public Health--Seattle & King County (PHSKC) to deliver the CM intervention, but not to enroll additional control participants. Therefore, between April 2008 and August 2008, we randomly assigned participants 3:1 to the intervention and control arms. We conducted analyses with and without the men enrolled under the 3:1 randomization scheme. Results were similar and we present the results of analyses that included all randomized men in this manuscript. While uneven randomization schemes may affect a trial's statistical efficiency, they have no impact on a trial's validity [25]. Follow-up ended in February 2009.

The same study personnel administered the CM intervention and conducted the study visits. These personnel also performed data entry of the results of the urine testing and HIV/STI testing. The behavioral endpoints were automatically transferred from the ACASI to a database without the potential for modification. It was not possible to blind those administering the CM intervention to a participant's study arm.

The University of Washington institutional review board approved the study protocol. All participants provided written informed consent.

### Study procedures

All study procedures took place at a large, public-transport accessible, community-based AIDS service organization. We screened participants for eligibility by phone and in person. All participants attended scheduled study visits every six weeks for six months. At each study visit, participants completed an audio computer-assisted self-interview (ACASI) that used a 6-week recall period and included questions about sexual behavior and substance use at the respondent- and partnership-level. Participants submitted urine samples for methamphetamine and cocaine metabolite testing at each of these visits.

At enrollment, 12 weeks, and 24 weeks, participants were tested for HIV/STI after completing the ACASI [26]. Participant-centered risk reduction counseling accompanied all testing [27], and condoms and lubricant were offered to all participants. Given the connections between substance use and sexual risk, HIV risk reduction counseling often included discussions of substance use.

### Study endpoints

The primary outcome was report of unprotected anal intercourse with a partner of unknown or discordant HIV status (non-concordant UAI) in the prior six weeks. The study included four secondary endpoints: the number of non-concordant UAI partners; results of methamphetamine urine testing; self-reported weekly or more frequent use of methamphetamine; and, self-reported use of >8 quarters (two grams) of methamphetamine. We chose non-concordant UAI as the primary outcome because this study was designed as a preliminary test of whether CM can be ultimately employed as an HIV prevention tool. We have previously shown this metric to be associated with HIV acquisition [28]. The secondary substance use outcomes represent intermediate variables in the causal chain in which methamphetamine use may facilitate the high risk sex that in turn leads to HIV/STI transmission.

### Reliability of behavioral study endpoints

We conducted a test-retest reliability study of the behavioral endpoints assessed in this trial [29]. From December 2006 to March 2007, we enrolled 102 MSM recruited from an STI clinic and an HIV clinic to complete the ACASI employed in this study. Of the 102 men who completed an initial ACASI, 98 (96%) returned to complete a second ACASI in a median of 4 days (range: 2-5). Participants took no more than 38 minutes to complete the initial ACASI. We calculated reliabilities using kappa statistics for binary outcomes and intraclass correlation coefficients by repeated measures analysis of variance (ANOVA) for continuous outcomes. The reliabilities of self-reported non-concordant UAI, weekly or more frequent use of methamphetamine, number of non-concordant UAI partners, and number of quarters of methamphetamine were 0.72 (95%CI: 0.47, 0.87), 0.88 (95%CI: 0.62, 0.99), 0.85 (95%CI: 0.78, 0.91), and 0.96 (95%CI: 0.94, 0.99), respectively.

### Statistical analyses

The primary purpose of this trial was to gather data for the design of a phase IIb or III clinical trial. The study was originally designed to follow 60 individuals. Assuming that 14% of participants would report non-concordant UAI at baseline, this sample size was chosen to provide estimates of the proportion of men who report non-concordant UAI in the prior six weeks with an expected width of the 95% confidence interval (CI) of 0.085. We expected to lose 25% of our study population to follow-up and aimed to enroll 80 participants. Additional resources, however, allowed us to enroll 127 participants.

We used chi-squared tests and *t*-tests to compare retention at the 24-week study visit and CM intervention adherence, respectively, between groups of participants defined by selected characteristics. We used generalized estimating equations (GEE) to estimate the proportion of participants reporting non-concordant UAI in the prior six weeks at each study visit. Analyses estimating the effectiveness of CM were intention to treat, on the basis of a binomial regression models with log links, to calculate relative risks comparing the proportion of men reporting non-concordant UAI between the study groups at study visits during the 12-week intervention and 12-week follow-up periods [30]. Our analyses of secondary binary endpoints employed the same analytic methods. Finally, we used a GEE model with a log link and negative binomial errors to evaluate the intervention's effect, expressed as a rate ratio, on the number of non-concordant UAI partners. We defined statistical significance at the *P *< 0.05 level.

We pre-selected baseline covariates that we thought would most strongly predict our outcomes of interest for adjusted analyses, including predictors of retention at the 24-week study visit. Of these covariates, we adjusted for variables that resulted in at least a 10% change in the adjusted RR (aRR) compared to the unadjusted RR [31]. We chose such strict criteria because of our relatively small sample size and because several potential confounders differed between our intervention and control groups. All analyses included a covariate indicating the baseline value of the outcome of interest. Estimates of the proportion of men reporting non-concordant UAI at each visit and analyses comparing any non-concordant UAI and number of non-concordant UAI partners between CM and control were also adjusted for HIV status and use of other substances (inhaled nitrites, gamma-hydroxybutyrate, ecstasy, or erectile dysfunction medications) in the prior 6 weeks at baseline. Analysis of detection of methamphetamine use by urinalysis was also adjusted for stage of change for methamphetamine use at baseline. We note that duration of methamphetamine use and injection use of methamphetamine were both evaluated as potential confounders; their inclusion in models that already included the above variables produced similar results to analyses without these two predictors.

We assessed participants' stage of change for methamphetamine use by a single question [32,33]: "Have you been trying to cut back or stop using methamphetamine?" This question was added after the first 19 participants were enrolled as it was not a variable we had initially planned to collect. The pattern of missingness, however, is missing completely at random (MCAR) which is unlikely to introduce bias to complete case analyses like the one presented in this manuscript [34].

## Results

### Enrollment, retention, and participant characteristics

**Figure 1:**
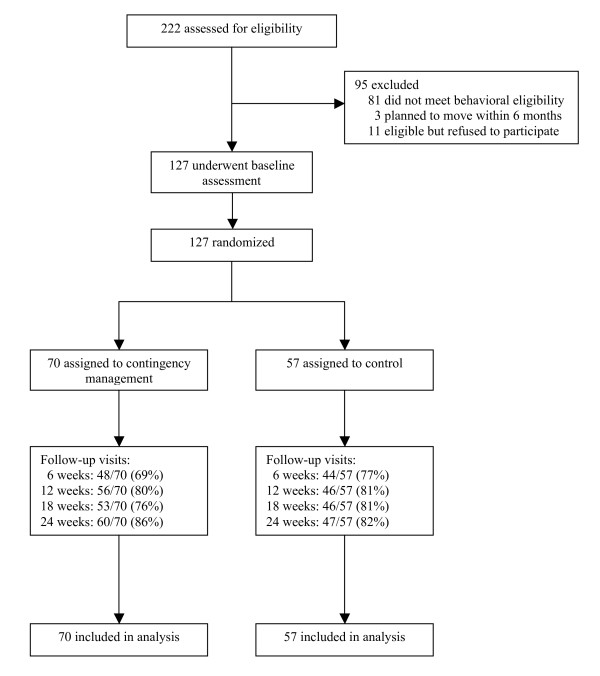
**Participant flow through the study**.

Over our 15-month enrollment period, we screened 222 men for eligibility (14.8/month) and enrolled and randomized 127 men (8.5/month). Of these randomized participants, 107 (84%) attended the 24-week study visit; retention at 6-week assessments was similar for both groups (Figure 1). HIV-negative men were less likely to attend the 24-week study visit than HIV-positive men (74% v. 93%, *P *< 0.01) and men who reported methamphetamine use for ≥10 years were less likely to attend the 24-week study visit than men who reported methamphetamine use for <10 years (81% v. 94%, *P *= 0.05).

The largest difference in follow-up between the CM and control group was for the first visit after baseline (69% v. 77%). Of the 22 CM participants loss-to-follow-up at this visit, one was incarcerated, one was in a drug rehabilitation program, three withdrew from the study, and 17 could not be located for study visit attendance. Among the 13 control group participants who did not complete the first follow-up visit, one had died, two withdrew from the study, and 10 could not be located for study visit attendance.

Table 1 presents characteristics of the study population. The only statistically significant difference between the study populations at baseline was that between the distributions of race/ethnicity (*P *= 0.03).

**Table 1:** Characteristics of the study population at baselinea

	Total Sample (n = 127)	Contingency Management (n = 70)	Control (n = 57)
**Recruitment**			
Peer-referral	64 (50)	35 (50)	29 (51)
Healthcare provider, STD or HIV clinic	28 (22)	15 (21)	13 (24)
Self-referral via community advertising	21 (16)	13 (19)	8 (14)
Community-based organization	7 (5)	4 (6)	3 (5)
Other, unknown	7 (5)	3 (4)	4 (7)
**Randomization**			
1:1	100 (79)	50 (71)	50 (88)
3:1	27 (21)	20 (29)	7 (12)
**Demographics**			
Age (years, median [range])	39 (18-60)	40 (18-60)	37 (19-57)
Race/Ethnicity^b^			
Black, African American	10 (8)	2 (3)	8 (14)
Hispanic, Latino	17 (13)	12 (17)	5 (9)
Native American, Alaska Native	8 (6)	4 (6)	4 (7)
White	76 (60)	47 (67)	29 (51)
Other race/ethnicity, multiracial, unknown	16 (13)	5 (7)	11 (19)
Education			
Less than High School	19 (15)	10 (14)	9 (16)
High School	30 (24)	17 (24)	13 (23)
Some college, vocational/technical training	60 (47)	35 (50)	25 (45)
College or greater	13 (10)	6 (9)	7 (12)
Missing	5 (4)	2 (3)	2 (4)
Annual income less than $15,000	90 (71)	53 (76)	37 (65)
Homeless or marginally housed	27 (21)	17 (24)	10 (17)
Neither employed nor attending school	89 (70)	51 (73)	38 (67)
HIV status			
Negative	57 (45)	30 (43)	27 (47)
Positive	70 (55)	40 (57)	30 (53)
Sexual orientation			
Gay, homosexual	80 (63)	47 (67)	33 (58)
Bisexual	28 (22)	14 (20)	14 (25)
Straight, heterosexual	1 (1)	1 (1)	0
Queer, same gender loving, other	18 (14)	8 (11)	10 (17)
**Sexual Behavior, prior 6 weeks**			
Number of sex partners (median [IQR])	3 (2-8)	4 (2-10)	3 (2-6.5)
Number of anal sex partners (median [IQR])	2 (1-5)	2 (1-6)	2 (1-5)
Number of nonconcordant UAI partners (median [range])	0 (0-35)	0 (0-35)	0 (0-16)
Nonconcordant UAI	35 (28)	21 (30)	14 (25)
Use of methamphetamine with anal sex	102 (80)	59 (84)	43 (75)
Exchanged sex for methamphetamine	20 (16)	11 (16)	9 (16)
**Methamphetamine Use**			
Duration of methamphetamine use, years (median [IQR])	11 (5-19)	14 (6-19)	11 (5-16)
Weekly or daily methamphetamine use, prior 6 weeks	83 (65)	51 (73)	32 (56)
Used more than 8 quarters of methamphetamine, prior 6 weeks	59 (46)	37 (53)	22 (39)
Injection of methamphetamine, prior 6 weeks	69 (54)	41 (59)	28 (49)
Trans-theoretical stage of change: "Have you been trying to stop or cut down on your methamphetamine use?"			
"Yes, I have been trying for more than 6 months" (Maintenance)	49 (39)	25 (36)	24 (42)
"Yes, I have been trying for less than 6 months" (Action)	24 (19)	12 (17)	12 (21)
"No, but I really want to start this month" (Preparation)	14 (11)	11 (16)	3 (5)
"No, but I really would like to try in the next 6 months" (Contemplation)	14 (11)	9 (13)	5 (9)
"No, and I am really not interested in trying" (Pre-contemplation)	6 (5)	3 (4)	3 (5)
Missing	20 (16)	10 (14)	10 (17)
Ever been in substance abuse treatment for methamphetamine use	32 (25)	17 (24)	15 (26)
Ever attended a support group for methamphetamine use	52 (41)	25 (36)	27 (47)
**Other substance use, prior 6 weeks**			
Cocaine	35 (28)	17 (24)	18 (32)
Crack	53 (42)	26 (37)	27 (47)
Inhaled nitrites	59 (46)	35 (50)	24 (42)
Erectile dysfunction medications	39 (31)	26 (37)	13 (23)
Ecstasy	19 (15)	8 (11)	11 (19)
Gamma-hydroxybutyrate	25 (20)	16 (23)	9 (16)
**Urine testing results**			
Urine positive for methamphetamine	44 (35)	28 (40)	16 (28)
Urine positive for cocaine metabolites	31 (24)	12 (17)	19 (33)
Urine positive for methamphetamine or cocaine metabolites	65 (51)	35 (50)	30 (53)

IQR, interquartile range; UAI, unprotected anal intercourse.

^a^Data are n (%) unless otherwise noted.

^b ^*P *< 0.05

### CM intervention adherence

Table 2 provides metrics of intervention adherence among CM participants. Participants who earned <$15,000 per year attended more visits than participants who earned ≥$15,000 per year (41% v. 20%, *P *= 0.04). Participants who reported using methamphetamine weekly or daily at baseline attended fewer visits than participants who reported less frequent use (31% v. 51%, *P *= 0.02) and participants whose urine contained methamphetamine at baseline attended fewer urine testing visits than participants whose urine did not contain methamphetamine (26% v. 43%, *P *= 0.02). Since the mean detection time of the methamphetamine assay is shorter than the interval between urine testing visits, the proportion of metabolite-free urine samples provided by CM participants during the intervention visits may be over-estimated.

**Table 2:** Contingency management intervention metrics

**Metric**^**a**^	**Participants (n = 70)**^**b**^
% Visits attended, mean (SD); median (IQR)	37 (1.4); 25 (8-54)
% Visits attended, n (%)	
0%	4 (6)
1-25%	32 (46)
26-50%	15 (21)
51-75%	10 (14)
75-99%	6 (9)
100%	3 (4)
% Metabolite-free urine samples out of those attended, mean (SD); median (IQR)	75 (3.6); 85 (0.5-1.0)
% Metabolite-free urine samples out of total possible visits, mean (SD); median (IQR)^b^	29 (1.3); 18 (8-42)
% Metabolite-free urine samples, n (%)^c^	
0%	7 (10)
1-25%	37 (53)
26-50%	13 (19)
51-75%	5 (7)
75-99%	6 (9)
100%	2 (3)
Number of continuous metabolite-free samples, mean (SD); median (IQR)^c^	4.2 (5.5); 2 (1-5)
Number of continuous metabolite-free samples, n (%)^c^	
Never submitted a metabolite-free sample	7 (10)
Submitted only single, non-consecutive metabolite-free samples	25 (36)
2-8	28 (40)
9-12	4 (6)
13-24	6 (9)
Earnings, US$ mean (SD); median (IQR)	112 (138); 50 (15-150)

IQR, interquartile range; SD, standard deviation.

^a^Data are n (%) unless otherwise noted.

^b^63 participants had 24 possible visits, 5 participants had 36 possible visits, 1 participant had 33 possible visits, and 1 participant had 25 possible visits (mean = 25 visits).

^c^We assumed that during visits the participant did not attend, he would have submitted a urine sample positive for methamphetamine or cocaine metabolites.

### Sexual risk

**Figure 2:**
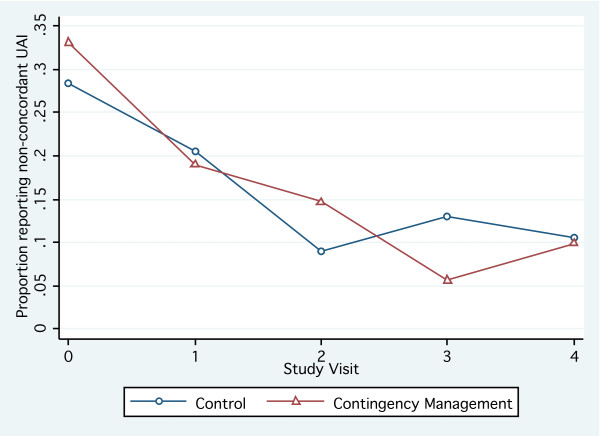
**Adjusted proportion of participants reporting non-concordant UAI in the prior six weeks, by study arm**.

At baseline, 6-weeks, 12-weeks, 18-weeks, and 24-weeks, the adjusted probabilities of reporting non-concordant UAI in the prior six weeks were 31.0% (95%CI: 27.6%, 34.3%), 19.7% (95%CI: 16.3%, 23.1%), 12.1% (95%CI: 9.9%, 14.4%), 9.1% (95%CI: 7.2%, 10.9%) and 10.2% (95%CI: 8.3%, 12.1%), respectively. Figure 2 presents the adjusted proportion of men reporting non-concordant UAI at each study visit by study arm. Comparing participants at study visits six weeks apart, participants at the later study visit were less likely to report non-concordant UAI in both the CM (aRR = 0.80; 95%CI: 0.64, 0.99) and control (aRR = 0.84; 95%CI: 0.71, 1.00) arms than participants at the earlier study visit.

CM and control participants were similarly likely to report non-concordant UAI during the 12-week intervention period. CM participants were less likely than control participants to report non-concordant UAI during the subsequent 12-week follow-up period, and also reported fewer non-concordant UAI partners than control participants during both the intervention and follow-up periods. None of these differences were statistically significant (Table 3).

**Table 3:** Effect of a contingency management intervention on reports of any non-concordant unprotected anal intercourse in the prior 6 weeks and the number of non-concordant unprotected anal intercourse partners in the prior 6 weeks

	Proportion reporting any non-concordant UAI, n (%)				
			
Visit	Total	Contingency Management	Control	Unadjusted RR (95% CI)	**Adjusted RR**^**a **^**(95% CI)**
Baseline	35/127 (28)	21/70 (30)	14/57 (25)		
Week 6	18/92 (20)	9/48 (19)	9/44 (20)		
Week 12	12/102 (12)	8/56 (14)	4/46 (9)	1.13 (0.54, 2.37)	0.80 (0.47, 1.35)
Week 18	9/99 (9)	3/53 (6)	6/46 (13)		
Week 24	11/107 (10)	6/60 (10)	5/47 (11)	0.67 (0.24, 1.85)	0.51 (0.21, 1.25)

	Self-reported non-concordant UAI partners, mean (SD)				
			
Visit	Total	Contingency Management	Control	Unadjusted Rate Ratio (95% CI)	Adjusted Rate Ratio^b ^(95% CI)

Baseline	1.35 (4.6)	1.70 (5.5)	0.90 (2.7)		
Week 6	0.47 (1.5)	0.35 (0.9)	0.60 (1.9)		
Week 12	0.19 (1.0)	0.09 (0.3)	0.30 (1.4)	0.46 (0.14, 1.52)	0.58 (0.17, 1.90)
Week 18	0.12 (0.4)	0.09 (0.4)	0.15 (0.4)		
Week 24	0.11 (0.4)	0.10 (0.3)	0.13 (0.4)	0.70 (0.25, 1.95)	0.49 (0.18, 1.37)

CI, confidence interval; RR, relative risk; SD, standard deviation; UAI, unprotected anal intercourse.

^a^Adjusted for HIV status, report of non-concordant UAI in the prior 6 weeks at baseline and use of other substances (inhaled nitrites, gamma-hydroxybutyrate, ecstasy, or erectile dysfunction medications) in the prior 6 weeks at baseline.

^b^Adjusted for HIV status, number non-concordant UAI partners in the prior 6 weeks at baseline and use of other substances (inhaled nitrites, gamma-hydroxybutyrate, ecstasy, or erectile dysfunction medications) in the prior 6 weeks at baseline.

One man in CM arm and one man in the control arm acquired pharyngeal gonoccocal infection (3.4 v. 4.5 per 100 person-years; *P *= 0.43). There were no incident syphilis, rectal gonococcal, or rectal chlamydial infections. Among the HIV-negative participants, one man in the CM arm and no men in the control arm acquired HIV (8.9 v. 0 per 100 person-years; *P *= 0.27).

### Stimulant use

During the 24 weeks of follow-up at the 6-week study visits attended by both CM and control participants, 28 (49%) control participants and 44 (63%) CM participants submitted ≥1 sample containing methamphetamine metabolites; 26 (46%) control participants and 23 (33%) CM participants submitted ≥1 sample containing cocaine metabolites; and, 43 (75%) control participants and 55 (79%) CM participants submitted ≥1 sample containing methamphetamine or cocaine metabolites.

CM and control participants were comparably likely to submit urine samples positive for methamphetamine at study visits during the intervention period (Table 4); however, during the follow-up period, CM participants were somewhat more likely to submit a urine sample containing methamphetamine (*P *= 0.11).

**Table 4:** Effect of a contingency management intervention on detection of methamphetamine use by rapid urine screen and on self-reported frequency and quantity of methamphetamine use in the prior 6 weeks

	Positive methamphetamine urinalysis, n (%)				
			
Visit	Total	Contingency Management	Control	Unadjusted RR (95% CI)	**Adjusted RR**^**a **^**(95% CI)**
Baseline	44/127 (35)	28/70 (40)	16/57 (28)		
Week 6	27/92 (29)	17/48 (35)	10/44 (23)		
Week 12	33/100 (33)	20/55 (36)	13/45 (29)	1.39 (0.81, 2.38)	1.09 (0.76, 1.57)
Week 18	39/97 (40)	24/52 (46)	15/45 (33)		
Week 24	39/105 (37)	29/58 (50)	10/47 (21)	**1.77 (1.13, 2.78)**	1.21 (0.95, 1.54)

	Self-reported weekly or more frequent methamphetamine use, n (%)				
			
Visit	Total	Contingency Management	Control	Unadjusted RR (95% CI)	Adjusted RR^b ^(95% CI)

Baseline	83/127 (65)	51/70 (73)	32/57 (56)		
Week 6	32/92 (35)	20/48 (42)	12/44 (27)		
Week 12	39/102 (38)	25/56 (45)	14/46 (30)	1.50 (0.93, 2.42)	1.29 (0.82, 2.04)
Week 18	37/99 (37)	26/53 (49)	11/46 (24)		
Week 24	40/107 (37)	29/60 (48)	11/47 (23)	**2.06 (1.29, 3.29)**	**1.76 (1.13, 2.73)**

	Self-reported use of more than eight quarters of methamphetamine, n (%)				
			
Visit	Total	Contingency Management	Control	Unadjusted RR (95% CI)	Adjusted RR^c ^(95% CI)

Baseline	59/127 (46)	37/70 (53)	22/57 (39)		
Week 6	22/91 (24)	14/47 (29)	8/44 (18)		
Week 12	28/99 (28)	21/55 (37)	7/44 (16)	**2.01 (1.09, 3.73)**	1.80 (0.95, 3.40)
Week 18	23/97 (24)	19/52 (36)	4/45 (9)		
Week 24	30/105 (29)	24/59 (40)	6/46 (13)	**3.52 (1.70, 7.30)**	**3.02 (1.47, 6.23)**

CI, confidence interval; RR, relative risk; one quarter of methamphetamine is equivalent to 0.25 grams. Statistically significant results (*P *< 0.05) are bolded.

^a^Adjusted for methamphetamine urinalysis result at baseline and stage of change for methamphetamine use at baseline (maintenance, action, preparation, contemplation, pre-contemplation, missing).

^b^Adjusted for baseline self-reported weekly or more frequent use of methamphetamine in the prior 6 weeks.

^c^Adjusted for baseline self-reported use of more than eight quarters of methamphetamine in the prior 6 weeks.

Comparing participants at study visits six weeks apart, control participants at the later study visit were less likely to report either weekly or daily methamphetamine use or using >8 quarters of methamphetamine in the prior six weeks than participants at the earlier study visit (aRR = 0.77; 95%CI: 0.68, 0.88 and aRR = 0.69; 95%CI: 0.56, 0.86, respectively); although the frequency and quantity of methamphetamine use declined over time among men in the CM arm, these changes were not as large with respect to frequency (aRR = 0.89; 95%CI: 0.83, 0.96) and not statistically significant with respect to quantity (aRR = 0.93; 95%CI: 0.85, 1.02). CM participants were significantly more likely than control participants to report weekly or daily methamphetamine use and to report using >8 quarters of methamphetamine at each study visit; these differences were statistically significant during the 12-week follow-up period (Table 4).

Accounting for baseline stage of change, control group participants were more likely than CM participants to report being in the action or maintenance stage of change for methamphetamine use during the intervention (74% v. 63%, *P *= 0.57) and follow-up periods (81% v. 66%, *P *= 0.18); however, these differences were not statistically significant. In the 6 weeks prior to baseline, 19% of control participants and 14% of CM participants participated in a substance abuse treatment program or peer support group (*P *= 0.45). After baseline, 26% of control participants and 21% of CM participants reported participating in such interventions (*P *= 0.38).

## Discussion

In this randomized controlled trial of CM, we enrolled 127 non-treatment-seeking MSM over 15 months and successfully collected follow-up data on over 80% of participants. These findings suggest that a randomized controlled trial that specifically seeks to enroll this population is acceptable and feasible. However, less than half of our participants were HIV-negative and restricting enrollment to HIV-negative MSM who use methamphetamine, the population of interest for a trial using HIV as an outcome, would be challenging. Unlike HIV-negative men, HIV-positive men are often enrolled in ongoing medical care and case management. HIV-positive men may also be more likely to recognize their methamphetamine use as problematic with some attributing their HIV infection to their methamphetamine use [35]. In addition, the success of peer recruitment (over 50% of HIV-negative participants were recruited through peers) over place-based recruitment strategies that have been successful in other HIV prevention trials [36] indicates that HIV-negative MSM who use methamphetamine may not share the same social and geographic spaces as HIV-negative men who do not use methamphetamine. HIV-negative men may have also been uncomfortable participating in a trial conducted at an organization well known for its services for HIV-positive individuals.

Unfortunately, our findings related to CM are discouraging. First, a relatively small proportion of men consistently provided urine specimens for the intervention meaning that actual exposure to the intervention among men assigned to CM was quite limited. Several factors may contribute to CM participants receiving such a low "dose" of the intervention, including the location, the magnitude of the incentives, time limitations, and monitored collection of urine samples. Anecdotally, many men reported not attending urine-testing visits because they knew their urine would test positive. This anecdotal evidence is supported by our data. Out of all of the CM visits attended, a median of 85% of urine samples were free of stimulant metabolites. Second, and most importantly, our findings suggest that CM is very unlikely to be effective as a stand-alone intervention among MSM. While the 95% CI around the intervention-period aRR was very wide and does not rule out the possibility that CM could reduce methamphetamine use during the period of actual intervention, our follow-up period aRR and 95% CI exclude the possibility that CM would have a large, sustained benefit on methamphetamine use as a stand-alone intervention in a population like the one we studied. In addition, the control group reported larger declines in self-reported use over study follow-up than the CM arm. Although measures of sexual risk among our CM participants were similar to or lower than those among control participants, it is unclear how CM might result in lower or similar levels of sexual risk if it increases methamphetamine use.

We are aware of six studies evaluating CM among individuals who use methamphetamine in outpatient substance abuse treatment [15,21,37-40]. Five of those trials found that, compared to other psychosocial interventions, CM leads to the submission of more stimulant-free urine samples and an increased duration of abstinence from stimulants during the CM intervention period [41]. None of the three studies that followed participants beyond the intervention period reported differential effects of CM compared to other treatments, but all three studies showed that all participants experienced sustained reductions in methamphetamine use for up to 8 months after ceasing to receive CM. Among MSM seeking outpatient substance abuse treatment, CM has been shown to be comparable in decreasing methamphetamine use and receptive UAI as other psychosocial interventions [15]. However, none of the previously published randomized trials included a no-treatment/minimal intervention control arm making it difficult to determine whether the absence of differences between CM and other interventions reflects comparable levels of efficacy or an absence of effect with all interventions.

Our study differs in four important ways from previous studies of CM. First, while we did not exclude participants who were receiving other substance use interventions, participants were not enrolled because they were seeking drug treatment. Second, we compared CM to a minimal intervention control arm. Third, CM was employed as a stand-alone intervention. Finally, CM participants were only expected to submit urines samples two times per week rather than three times, as has been done in some, but not all studies of CM [21]. We cannot say whether CM's failure to reduce methamphetamine use in our study reflects a lack of effect among persons who are not expressly seeking drug treatment, CM's ineffectiveness as a stand-alone intervention, the ineffectiveness of our CM schedule, or a more general lack of efficacy of CM that would have been consistently observed in other trials had those studies included no-treatment/minimal intervention control groups.

We found that self-reported methamphetamine use declined among control participants but remained relatively stable among CM participants. We cannot readily explain this effect. We do not believe that CM participants used vouchers to obtain methamphetamine, since the difference in drug use by study arm was only evident after CM recipients ceased to receive vouchers. Likewise, we do not believe our findings simply reflect more accurate reporting of methamphetamine use by CM participants since we found that CM recipients were also more likely to test positive for methamphetamine. Participants in the two study arms reported similar use of outside treatment and support services, making it unlikely that differential use of other treatments affected our results. It is possible that participation in the CM arm may have put participants in contact with other participants and CBO clients in ways that facilitated access to and use of methamphetamine. Similar phenomena have been reported in other studies [42,43]. Also, discontinuation of twice-weekly CM visits, which may have provided a source of support for CM participants, may have led to heavier methamphetamine use among CM participants compared to controls.

Similar to a study of CM and other psychosocial interventions in the substance abuse treatment setting [15], we found that sexual risk declined in both of our study arms. However, in the case of the current study, our comparison arm was a minimal intervention control group. People who volunteer for studies may be more motivated to change their behavior than those who do not, participants may enroll during a period of high-risk activity and decrease their risk independent of intervention effects, and simply measuring a behavior may change a participant's behavior or reports of that behavior [44]. Each of these explanations may contribute to why sexual risk in our study, as in past studies, declined in both control and CM arms. Had we not used a minimal intervention control group and only measured participants in the CM intervention, we might have erroneously concluded that CM reduced sexual risk. This finding emphasizes that no-treatment/minimal intervention control groups are essential to rigorous intervention evaluation.

We note that the characteristics of our study population and the outcomes among CM participants were generally similar to those in a San Francisco public health program designed to reduce methamphetamine use among MSM (PROP) from December 2003 to December 2005. Our screening (14.8/month in the current study and 10.3/month in PROP) and enrollment rates (8.4/month and 7.4/month), attendance at CM visits (37% and 41%), and incentives earned (24% and 31% of the total possible) were similar [17]. Compared to PROP participants [18], participants in the present study were more likely to have used methamphetamine for >10 years (50% v. 38%), but were less likely to report weekly or more frequent methamphetamine use (64% v. 86%) and to be HIV-positive (55% v. 78%). More than 80% of men in both populations reported methamphetamine use with sex and 54% of participants reported injecting methamphetamine at enrollment. These similarities demonstrate that our trial probably closely replicated what might occur in a non-research setting.

Our study has several important limitations. First, we did not power the current study to detect differences between study groups based on methamphetamine use or sexual risk. Instead, this study was powered to provide precise estimates of the proportion of methamphetamine-using MSM who report non-concordant UAI. Despite this limitation, the precision of our post-intervention period aRR rules out the potential of CM to effect a large, sustained reduction in methamphetamine use as a stand-alone intervention, at least in the population we followed. Second, randomization did not provide us with study arms balanced with respect several pertinent variables. In particular, persons assigned to the CM arm were more likely to use methamphetamine at baseline, and higher levels of drug use have been associated with inferior responses to CM in prior studies [45]. It is possible that our results are subject to confounding bias stemming from imbalances in variables that we did not measure. Third, we altered the CM intervention after beginning the study. These modifications may have not provided sufficient incentive for abstinence from methamphetamine use [20]. Finally, our measures of self-reported sexual behavior and substance use are subject to social desirability bias.

## Conclusions

In summary, our small, randomized trial found that a 12-week CM intervention was associated with a potential increase in methamphetamine use, but decreases in sexual risk that were not statistically significant. While our experience suggests that a larger, more definitive controlled trial of CM to decrease methamphetamine use and sexual risk may be feasible, our findings suggest that CM would be unlikely to effect large, sustained reductions in methamphetamine use among MSM.

## Competing interests

The authors declare that they have no competing interests.

## Authors' contributions

TWM participated in study design and coordination, performed the statistical analyses, and drafted the manuscript. DRJ participated in study coordination. JPH participated in the design of the study and provided statistical expertise. GNC and SS participated in the conception and design of the study. MRG conceived of the study and its design and helped draft the manuscript. All authors contributed to the interpretation of the study data, revised it critically for important intellectual content, and read and approved the final manuscript.

## Pre-publication history

The pre-publication history for this paper can be accessed here:

http://www.biomedcentral.com/1471-2458/10/774/prepub
